# Artificial Nourishment of Sucklings in the First Ten Weeks of Life

**Published:** 1881-02

**Authors:** 


### ARTIFICIAL NOURISHMENT OF SUCKLINGS IN THE FIRST TEN WEEKS OF LIFE.

THIS subject has lately been attracting a great deal of attention in Germany, and a special committee has been appointed by the scientific societies to investigate it, one of the first fruits of which is this article from Dr. Kormann. We have already published an extract of an article by Dr. Dornblüth, and the present one does not bring up any very particularly new points. The author starts with the importance of urging upon mothers the importance of nursing their own children, and, where that is impossible, getting a good wet-nurse. These two failing, he makes a third point, that no starchy substances should be given in the first ten weeks, but that our reliance must be on the milk of domestic animals. Mare's milk he claims is the best, but this being hard to obtain, we must usually fall back on the cow. This branch of the subject he divides into two parts: 1st, How to obtain a cow's milk which is fitted to the nourishment of a suckling; and 2d, How to treat such a milk in order to put it in the best form and consistence for use.

Under the first head are two points: 1st. We must have the best quality of milk; and 2d, This must be delivered unaltered at the house where it is to be used. To attain the first requisite, he would have in each city dairy-farms under strict governmental, veterinary, and medical control. The best races of animals should be selected, they should be kept in stall and fed on regulated diet, and carefully watched. Under the next head he has numerous projects, of which the best seems to be that the milk, obtained at the farms under this strict control, should be sent in locked flasks to the consumers, and that they only should have the keys to these flasks. Great precautions should be taken to insure cleanliness, not only of all vessels used, but of the hands of the milkers, the cows' udders, etc. He discusses Dornblüth's plan of treating the milk by cold, and claims that it is good, but seems to think more of a plan of heating the milk in the sealed flasks, and so delivering it. There already are a few institutions in Germany where this carefully-watched "children's milk" is sold, and the author claims a wonderful immunity in such districts from summer diarrhœa, etc. The use of such milk is principally a matter of cost, but even with the increased cost over milk it is cheaper than the various less useful surrogates.

Under his second principal heading are two divisions: 1st, How to take care of the milk after it reaches the consumer; and 2d, How to treat it to make it most closely resemble woman's milk. Under the first head, he believes in boiling the milk as soon as it reaches the consumer (a point in which he would be opposed by some good authorities). He claims that this at least avoids all micro-fungoid growths. After boiling, it should be kept in good, cool air, and in tightly closed vessels. All vessels used, especially the nursing bottle and its nipple, should frequently be carefully cleaned with solution of soda. As to the second point, we must first regulate the temperature to make it as nearly as possible that of the human body. We must also add water and sugar, and attempt to correct the clumpy coagulation of the caseine. Jacobi's barley water he claims is good, and he uses it in the following proportions: In the first 4 weeks, 1 pint milk : 4 pints barley water; from 5th to 8th week, 1 : 2; from 8th to 12th week, āā. To each 100 grm. of milk mixture, he adds a teaspoonful of good cane sugar, preferring this to the milk sugar, which is apt to become sour. He mentions Sulzer's albumin pepton and gum arabic, gelatine, etc., as used for the same purpose as barley water, but has had no practical experience in them. Then he discusses lactin, or lacteine, and approves of it.

When good "children's milk" from the cow is not to be had, he sometimes uses condensed milk, but prefers Biedert's cream mixture. In those cases in which, on account of diarrhœa, no milk diet can be used, he advises mucilaginous drinks, egg preparations, and especially Biedert's "cream-conserve," prepared artificially without a trace of real milk or cream.—*Jahrbuch f. Kindhlkde.*—*American Journal of Obstetrics.*

